# Reclaiming agency: skills, academics and students in the Social Sciences

**DOI:** 10.1057/s41304-021-00349-3

**Published:** 2021-09-28

**Authors:** Maxine David, Heidi Maurer

**Affiliations:** 1 Leiden University, Leiden, The Netherlands; 2 Danube University Krems, Krems an der Donau, Austria

**Keywords:** Academic skills, Active learning, Agency, Employability, Social sciences

## Abstract

The adoption of active learning pedagogy and, later, the institution of the employability agenda in Higher Education have resulted in a severe loss of agency for academics and students in the Social Sciences. In this article, we reflect on our experiences of applying active learning methods. We argue that we have been part of a change that has occasioned a loss of key skills development, especially those associated with traditional learning and academic thinking. An overly headlong rush to implement the “new” over the “old” saw the discarding of certain skills central to the active learning agenda. Further, the emphasis on student satisfaction, professionalisation and quality assurance pushed the academic to the sidelines, to the detriment of Higher Education. We, therefore, first critique the skills debate and identify shortcomings in the active learning application that emerged from that debate. We focus on the skills emphasised in practice, how they are portrayed in opposition (instead of complementarity) to academic skills, and how they undermine the agency academics and students really require. Next, we propose a reconsideration of necessary but undervalued skills like reading, listening and note-taking.

## Reflections on the employability and skills agenda in the Social Sciences

Change in education is necessary and to be expected. However, sometimes the nature of change is so radical as to constitute a rupture. The consequences of such a moment may be severe, disruptive and damaging. In such times, foundations can be lost. In this article, we argue that the employability agenda instituted in Higher Education (HE) constituted such a moment. However, it was facilitated by an excessively narrow interpretation and application of active learning pedagogy. The employability agenda premised a confining set of skills for employment, privileging active skills and defining them in such a way as to exclude other related, foundational skills. Further, the employability agenda has been particularly detrimental in being both caused by and a reflection of the rise of the student as consumer (Bunce et al. 2017). As a result of the borrowing of active skills from the natural sciences, especially medicine, there has been a discursive linking of employability or skills on the one hand and innovation via active learning on the other hand in the Social Sciences, too.

Subsequently, a fallacious understanding of active learning in the Social Sciences has developed. Two things can be said about this. First, it constitutes a blind disregard for the notion of universities as sites to develop critically questioning, reflective thinkers. Second, it suggests such thought is not itself a skill of utility in a wide range of careers and professions (and for citizens). Paradoxically, this overly exclusive interpretation of “active” in active learning and the continued construction of structures to propel excellence in learning has led to exactly the opposite in those geographies where active learning has been more deeply embedded. In the creation of administrative and bureaucratic structures at European (at least) universities, agency was relocated from the classroom to the boardroom. Deans’ offices, executive boards, senates etc. of universities increasingly provide strict requirements about the minimum/maximum number of assessment items, word count, age of texts to be relied on, educational tools to be used (Microsoft PowerPoint, lecture capture, reading list management systems) and number of sources to be used. All this leaves course convenors to *implement but not decide* what might best facilitate students´ learning. This article asks critical questions about what this loss of student and staff agency means for universities as places for building and practising academic thinking.

Before that, some acknowledgements. We, the authors, were ourselves persuaded by the turn to innovative teaching methods, focussed on active learning. Until more recently, David’s teaching was characterised by a rejection of wholesale traditional transmission-orientated teaching, tipping the balance in favour of enquiry-based learning (EBL), including problem-based learning (PBL), small scale investigations and research. Her application was and is directed at modules or parts of modules, as opposed to the more structured and systematic PBL approach that is the hallmark of Maastricht University, where Maurer taught from 2008 to 2017. There, students learned in the more structured form, using the “seven jump” approach (Maurer 2015). Based on our long experience in the classroom and reflections upon it, as well as our engagement with the wider academic community, this article acts as a cautionary tale for the academy in those geographies where the employability turn is being considered or beginning to be implemented. It offers, additionally, a launch platform for a critical discussion in the profession to reflect upon what has been lost by this turn. What we argue for is a reconsideration and rebalancing of the traditional with the innovative.

The time is particularly apt for such critical reflection given the appetite the COVID-19 pandemic has created to reconsider what we teach, how and why. Education has been elevated to the top of political and societal debates as “teachers” have been recognised as “frontline workers”, but also, we argue, in terms of their role as experts responding to societal and political developments (Harrison and Luckett 2019; Irani 2021). Equally, the pandemic has also cemented the understanding of learning as a social experience with the role of academics in this micro-society as one that has to be carefully navigated. What we deliver in this article, therefore, are ideas about when academics in the classroom should centre themselves and when they should centre the student. Crucially, however, we argue that *both* must happen.

Drawing on the conceptual unpacking of “skills” and “agency”, we show that the academic has gradually, in many contexts, suffered a severe loss of agency. But while many academics rail against the student as consumer, what is perhaps less understood is that the student has *also* lost agency. Both have lost their sense of place in HE (academics benefiting from their longer service in universities may be most aware of this). What is needed is a purposeful re-integration of the useful aspects of the old with the added value of innovation. What is also needed, though, is a return of the academic to the academy as expert and educator and the student as student, with ownership over their learning but not their teaching.

We first introduce the wider HE discussion on employability and skills. Next, we assess the reasons why active learning pedagogy has been transposed to the Social Sciences. We outline our critique of what has been lost in the rush to fix disciplines that were not broken. In a setting of “professionalised” teacher training, all-university educational development and increased quality assurance frameworks, academics in the Social Sciences have not mounted sufficient defences, have not taken the place they could have taken in the educational avantgarde and, as a consequence, have suffered from an attendant loss of confidence. The next part emphasises the skills lost in the rush to institute an employability agenda that has, largely, been about innovation in teaching, the outcome of an impression that teaching in HE hitherto was not fit for purpose. We focus on reading, listening and note-taking and treat all of these as foundational, active contributions to learning. We show that the preoccupation is with a flawed understanding of what constitutes “active” in the learning process. We also contend that the *active* aspects show too little consideration for outcomes in respect of *learning*. We deliver our own experience-based thoughts about the process through which learning takes place and argue this process *itself* reflects the development and employment of transferable skills. Again, we argue for a re-integration of basics. In the final part, we advocate an understanding of foundational skills. Bureaucratic processes and quality assurance have their place but the academic must be an active part of those processes, not the passive recipient of them. In short, we call for academics to reclaim their central place in the academy.

## Skills in the university

What should students take away from a university education and especially from a Social Science degree? This is a long-standing debate in academia and beyond, not only since the demands put forward by proponents of the Bologna process. “Employability”, often also labelled as “real-world” or “practical” skills, is a frequent buzzword in university administration. This focus on the direct translation of a university education into employment applicability also features highly in the European policy setting, such as in the 2005 overarching framework for qualifications termed “Dublin descriptors” adopted by the European Higher Education Area (2005; see also Maurer and Mawdsley 2014). But should academics act as mere trainers for employers? And in developing skills, do academics have nothing to say about what constitutes a vital skill?

In consulting studies about the employer perspective, we see more nuance than the employability debate in universities would lead us to expect. In a comprehensive study on employers´ perspectives on employability and skills in HE, Humburg et al. (2013) found that for graduates to be invited to a job interview, familiarity with the subject field, good grades and experiences that signal openness and independence (like studying abroad) are universally considered the most salient indicators by employers across fields. In relation to skills, they show “the most important skills are professional expertise and interpersonal skills” (2013: 94), that expert knowledge can be trained on the job, but this requires employees who have the capacity to acquire new knowledge and learn (2013: 75). Other parts of the debate reflect the situation in which one is applying the definition. In an article about employability in the UK, for instance, Morley (2001: 131) contextualised the “commodification” of the academy through reference to the closer relationship of academia to government policy and corporate interests. Morley additionally directed us to another issue in asking: “Do universities exist simply to meet the needs of modern capitalism and are students being constructed solely as future workers, rather than fully rounded citizens?” (Morley 2001: 132).

Before addressing this important point about citizenship, there are questions to be asked about *which* future job market we should be preparing students for and when, given signs that people will have more than a single career trajectory in the near future (Slade 2017). It is also noteworthy that skills are only very latterly being revisited in a background of technological changes that see shorter attention spans, the challenges engendered by an era of social media, fake news, disinformation campaigns and consequences for critical thought and vulnerability to malign intentions (Harrison and Luckett 2019). Thus, generally, the debate on employability has been interpreted narrowly, with assumptions made about what constitutes the “basics”. In addition, the linking of the skills agenda to employability (as a consequence of the liberal commodification occurring in many countries) rather than citizenship has drowned out significant conversations about the role of the university in creating active, reasoning citizens.

That said, this conversation is being held in certain places.^1^ The American Association of State Colleges and Universities has its American Democracy Project^2^ focussed on the nexus of HE and an informed citizenry. In the European context, publications listed on the Council of Europe’s website^3^ and articles such as those of van Mol (2018) or Biesta (2009) reveal similar concerns. Neither author can remember working in a university where this aspect of HE achieved anything like the amount of attention that the employability agenda does. Thus, while the public purpose of HE in educating citizens for democracy does feature in many arenas, these nuanced arguments do not spill over to dominate debates within universities themselves.

There is plenty of scope, however, to extend existing conversations, and the anti-intellectualist tendencies that have dominated in the “fake news”, populist rejection of expertise and pandemic contexts constitute an imperative for this to happen. In the UK, the University of Glasgow and Edge Foundation produced a comprehensive study that examined employers’ perceptions of new graduates that found “universities need to equip graduates with ‘deep’ intellectual capabilities and a battery of applied practical skills which make them more ´work-ready´” (Lowden et al. 2011). Intellectual capabilities, in this context, should not only mean that students are able to apply academic knowledge, but also that they understand the distinct nature and added value of academic thinking. This is consistent with the utilitarianism versus intellectualism dichotomy identified by Morley (2001: 132) but fits with our own pedagogical ethos, i.e. that HE does not need to be about one or the other, and that good instructional design can ensure that students’ encounters with learning are intellectually demanding and satisfying but simultaneously also develop skills relevant to a range of careers. In so doing, we also reject any idea that the majority of employers look at graduates for either purely utilitarian or purely intellectual purposes. After all, if skills are all that are required, why look for graduates versus apprentices?

The Social Sciences are historically well-equipped to balance that utilitarian-intellectual agenda but have, perhaps, lost sight of what they have to offer in debates about “necessary” methods and skills. What is needed, is a more careful reflection on what the Social Sciences have long done but also what needs to be added anew, for example, web literacy skills that are based on identifying reliable sources but also require more technical understanding (see Caulfield 2018). Universities need to assert what they undoubtedly are best-suited to provide, that is teaching students to think academically: to enquire systematically and critically, to make discerning decisions regarding what constitutes well selected evidence, and to be able to problematise and formulate well-defined questions. This stands in stark contrast to the way university administrations, especially in the UK, seem to think. The most recent example is the University of Sunderland which, in early 2020, justified the closure of its politics degree on the basis of its newly developed “career-focused and professions-facing approach” (University of Sunderland 2020). More widely, as so powerfully argued by Bulaitis (2021), the UK has seen government continue to laud STEM subjects, to the detriment of the Arts, Humanities and Social Sciences, on the basis that HE needs to be directed towards those subjects the Government has decided address “labour market needs”. Hence, at this point, it is also worth defending the idea that intellectualism itself does not preclude skills, rather academic thinking and academic working in the Social Sciences *constitute* skill sets for students (and employers). It is, therefore, only *sometimes* possible to separate the utilitarian from the intellectual agenda.

## The adoption of active learning for skills development and our critique

Employability and skills have been used as an argument by universities and staff alike to tap into active learning pedagogy: “active” students learn better, they enjoy learning more because they feel in charge and they learn skills along the way. The teaching and learning scholarship is almost universally positive about the benefits to students of adopting active learning methods (Deignan 2009). Barriers to a move from traditional methods to EBL or PBL are well-explored, as are some of the pitfalls in actually making the move (Deignan 2009). Despite the nuances identified in the literature, the way active learning pedagogy is adopted across many European and UK universities is suggestive of an acceptance that traditional teaching methods are no longer fit for purpose. In such attempts to innovate, the main emphasis is put on active learning as positive for students because they develop skills that are relevant for their future employment. But do traditional teaching methods really have so little to offer or are we guilty of throwing the baby out with the bathwater?

**Fig. 1:**
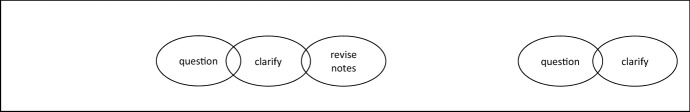
Skills featured in active learning approaches

Before that, what *is* active learning and what are its relative benefits? Active learning puts students into an active researcher role, emphasising the relevance of enquiry over factual knowledge. Learning is “a process of knowledge construction” (Glaser 1991: 131) rather than knowledge transfer. Methods include use of simulations, role plays, flipped classrooms and reflective learning in teaching and assessments, on the basis that students learn and internalise more if they are active participants in learning rather than passive recipients of knowledge transfer. Thus, active learning “refer[s] to an assortment of techniques where students do more than simply listen to a lecture” (Ishiyama 2013). In active learning environments, students work in small groups, supported by the academic as facilitator. They pose questions, clarify and revise their notes (see Fig. 1). Positive outcomes identified are: the emergence of more independent learners who are simultaneously better team-players and likely to be lifelong learners; the acquisition of problem-solving skills and other transferable skills; more critical thinkers (Deignan 2009).

Those are the benefits. What, however, are the lacunae or problems that we identified in our work as instructors in active learning environments? The first comes in relation to skills and our focus is on three skills: reading, listening and note-taking.^4^ Reading is, at least implicitly, covered in the active learning literature, although not sufficiently emphasised as a discrete, foundational skill. The others do not feature as key to learning. Thus, we contend that these three skills are assumed: all students know how to read, listen and take notes to the standard needed in tertiary education, in their specific discipline and particularly in an active learning environment. The active learning literature speaks of skills that we train in active learning but does not put sufficient emphasis on the underpinning skills needed to maximise the potential offered by active learning approaches. We offer a corrective by advocating early development of these three skills as fundaments of active learning methods.

Second, active learning methods are often portrayed in *opposition to* traditional methods of teaching, a corrective for the problems associated with “traditional” methods, which, at times have been equated to an “old-fashioned” and also “authoritarian” approach (Deignan 2009: 20). In earlier teaching days, both authors had sympathy for such arguments. To some extent, however, our experiences have caused us to inject more nuance into our thinking and practice. We reject the binary of the traditional versus the innovative, arguing that traditional methods honed key skills in a way that has been undervalued in active learning but which would greatly enhance its outcomes. In a later section, we further outline how the typical characterisation of the traditional as passive, and the innovative as active, is to deny the activity of the student when reading, listening and note-taking.

The third negative is that the replacement of traditional methods with an active learning environment has led to the problematic consequence that (1) agency is (fallaciously) perceived to shift from the educator to the student; and that, relatedly, (2) there is a false perception of student agency. Putting students in the driver's seat of enquiry without the right (skills) background equates to giving them a licence to be in charge of a vehicle (their own research process) without first offering adequate driving lessons.

Finally, in our critique of our own teaching and how we ourselves used the literature, we latterly realised that much of the active learning thinking emerged from scholarship largely focussed on medical, technical or natural science education. As a result of an unreflective adoption of these ideas and especially of the need to think about “employment skills”, academics in the Social Sciences were not properly appreciative of the fact that such skills had been integral to Social Sciences education for decades already (albeit those traditional skills were taught more by implication). Given our expertise lies in Politics and International Relations, this was doubly perplexing: why focus on employment-related skills in a discipline that already positively required (even if not always perfectly) students to acquire those self-same skills (e.g. research skills, communicating complex ideas orally and in writing, applying theory to current events)?

Our experience led us to conclude that losses associated with employing active learning approaches resulted from a failure to unpack that literature and engage in advocacy from the very start. It is important to emphasise, however, that we neither reject active learning nor advocate a return to purely traditional methods. Rather, we argue for a re-evaluation in how we adopt active learning pedagogy and for a hybrid approach that borrows from both the traditional and the innovative. A conversation, in a *non-dichotomous way*, about skills is warranted—and long overdue.

## Necessary but undervalued? Learning actively through reading, listening and note-taking

**Fig. 2:**
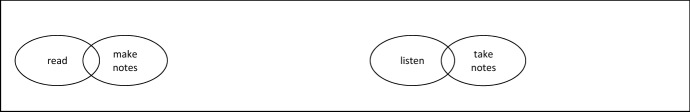
Skills featured in traditional teaching methods

Our critique of the adoption of the active learning literature is threefold: first, the foundational skills of reading, listening and note-taking (see Fig. 2) neither take a prominent role in teacher training nor feature prominently enough in the hands-on active learning resources. Both focus on the development of other skills when learning in an active manner rather than on those skills that are a *prerequisite* for active learning to work. Second, active learning methods are often portrayed in *opposition to* traditional methods of teaching. Third, we identify a loss of agency for both students and academics when the active learning pedagogy is applied superficially, and the employability discourse followed blindly.

Before exploring the foundational skills, a word on context. The problems identified above are exacerbated by other HE developments, especially technological. Lecture capture, posting of lecture slides, and linked library lists among others all conspire against active learning. This is not to say technology, when carefully applied, is not useful for building and developing academic thinking and skills. But if introduced in a blanket fashion, without the right pedagogical justification, these technological tools not only promote passive learning, they close down opportunities for skills development and deprive students of key, serendipitous means of learning. For instance, what did previous generations learn from spending time in stacks or databases that our students no longer learn because of the increasing tendency to use linked library lists or give overly concise (or extensive) reading lists? Whether willing or not, academics and students are the subjects of structural change that is driven by agendas other than the pedagogical one.

### Reading

Before turning to the literature, we counsel the reader to engage in some reflection on your training for teaching (we acknowledge access to this is not universal). Most likely, you followed some training about building students’ presentation skills, on how to engage students or assessment methods. But did anyone ever talk about how to get students to read in different ways and for different purposes and why reading is important? In the world of the professionalised academic, it is curious that teaching students the basic skills does not figure on the curriculum of what is taught to *us*. Is this simply because it is deemed unnecessary?

There is also the matter of what salience is given to foundational skills in academic “how to” handbooks for students. In a preliminary search for literature that speaks about the importance of reading skills, we found a good deal of work, but much of it old (Preston and Botel 1952; Kirby 1988). Much is also focussed on literacy versus the skill of reading. This suggests that as the battle for literacy levels was won, less attention was paid to reading as a skill that needed to be developed beyond the mere capacity to read (MacLellan 1997; for a good example see Kirton and McMillan 2007). Directed at secondary education, Duffy’s work (2009) is still relevant for the tertiary level, speaking of teaching students to read for a purpose, of bringing texts regularly into the classroom, of reading with the students and of finding texts that motivate them. MacLellan (1997: 277) affirms that increasing numbers of HE students create efficiency pressures that give students and academics less time to work together and, consequently, students are required to read more in self-study. This fits with the authors’ experiences, where we both provide extensive reading lists that our institutions require but work in semester or even block-long structures where we have little or no time to engage in a detailed discussion of a key text with students and instead, too often, relegate this to the student’s room rather than the classroom.

Most academics would likely recognise that we expect students to be able to engage with the literature independently, but it is *our* contention that reading needs to be elevated to the level of writing, “for reading and writing are not […] mere vehicles for gaining access to and transmitting information, but […] real epistemic tools that foster the construction and transformation of knowledge and, in consequence, the transformation of the mind” (Mateos et al. 2007: 490).

One final point. The act of reading may be passive but the thinking and learning around it are not, or at least should not be (see Tompkins 2016 for deeper guidance). Reading is about working with the text, critically engaging with the authors and their arguments, about contextualising the text. Reading is not about providing quotes and copy/paste snippets of text. And it is not about a mere search for textual passages that would help with a question developed in an active learning mode. In both traditional and active learning settings, space thus needs to be made in the classroom for discussing reading and for making sure students understand how central reading is to other tasks they perform. Opportunities to do this can be provided in a variety of exercises, where reading is both central and active in nature, students understanding that in order to deliver persuasive arguments, they first have to read.

Whether or not students *hear* such arguments is another matter, which brings us to the second of our skills: listening.

### Listening

There is this misperception that in contrast to active learning where students *do* things, listening is passive. That students listen “passively to an instructor’s lecture” (Faust and Paulson 1998: 4) is a common description of traditional learning that active learning tries to move away from. Or take the active learning definition above—that “*students do more than simply listen to a lecture*” (Ishiyama 2013). This misperception has been part of the cause for denigration, even dismissal, of the traditional lecture. Much, consequently, has been lost. And such unreflective binary categorisation misses the point, as lectures can be done well or badly, and being able to engage with a lecture requires being educated as to how to do that—and encouraged to do so. Listening is a “multidimensional construct” of understanding, stimulation and responding and thus combines cognitive, affective and behavioural processes (Gearhart and Bodie 2011: 86). In other words, listening requires students to process actively what they hear (Chen 2019). Fostering note-taking is one way to cement that. Afterwards, students can be further prompted to engage with what they listened to, e.g. through seminars where they articulate what they heard and compare reflections with their peers, which itself demands more active listening.

Connectedly, the lecture hall provides a space in which students are required to focus, in an educational experience too often crowded with the requirement to do many things at the same time. Where, in the active learning environment alone, do we send the message to students to listen, not to talk; to reflect inwardly, not outwardly? Importantly, we do not suggest these other things should not happen, just that space is needed to help students understand the discrete parts of the learning process. Studies show today’s multitasking environment has a detrimental effect on the ability to focus and to remember: “when our attention is divided, it becomes much more difficult to transfer information from our short-term memory, which is just the very temporary store, to our long-term memory, which is the seat of understanding” (Carr in Parry 2010). The lecture theatre can be a safe space in which the grounds for a conversation with students are first established. It proceeds more slowly and in a more directed fashion than a seminar, a space in which academics can have more initial control and learn to let go of that control at a pace that suits them and the student cohort. We, therefore, advocate teaching methods that build students’ capacity to focus, to listen and to internalise knowledge and understanding for the longer term.

Lectures have additional benefits. They situate the lecturer as a role-model of how to engage with academic scholarship. They serve as *examples* of how to synthesise knowledge and ideas—*if* students are given the opportunity to hear them and reflect on them in these terms. This could be done by role-modelling (the academic showcases to students how to engage with and reflect on a guest lecture) or by carefully integrating lecture content into future course activities (giving students opportunities to reflect on what they took away in the next seminar or in their journaling). Furthermore, lectures establish the academic as the expert, a not-unimportant issue in the “market” of education and a world where the student is a consumer. This is not solely about protecting the academic, it is about ensuring students have encounters in which they can see what it means to hold the stage, to convey complex ideas in an accessible but not over-simplified form. The dual opportunities here should not be underrated: (1) for the academic to hone and establish their expertise and authority; (2) for the student to have illustrative encounters with expertise that they can rely on to develop their own, authoritative, public voice. Such opportunities are particularly relevant in circumstances where academics and students encounter discrimination, especially in an era of increased anti-intellectualism and rejection of experts. These are related points that are not easily separated for they all speak to the fact that expertise matters and should be showcased.

In their study among medical professionals, Haidet et al. (2004) found medical residents’ *perceptions* were that the lecture was more valuable than active learning methods. The authors’ conclusions suggest perceptions of expertise are formed where expertise is most visible. There is a performative (which is not to deny the substantive) element to the lecture that should not be ignored. In dismissing the lecture as a place where academics take centre stage, we have forgotten other aspects of performance—that a good performance (lecture) can affect those in the audience in such a way as to draw them in, to feel a combined sense of aspiration and inspiration. Learning activities in which the academic is always relegated to the sidelines impact on perceptions of their expertise. And let us not forget the wider environment in which academics are working. “About half of all UK academics have suffered depression, anxiety or other types of mental health problems related to stress—one of the highest rates of any sector” (Grove 2018). Additionally, evidence shows women and minority scholars are likely to experience discrimination from students (Lilienfeld 2016). Some thought must be given to how these challenges can be mitigated. Clearly, a forum is needed through which academics, no matter their seniority, confirm their command over their field. The lecture serves this purpose well (as recognised by the existence of the professorial inaugural lecture).

And to return to the duality of this opportunity, this is about students as well. By restoring academics to the lecture hall setting, they can serve as living, necessary examples for those students who may face issues with establishing authority in the face of discrimination, whether within the university or beyond it.

By moving to a culture of predominantly active learning and away from lecturing, we contend that more has been lost than has been properly appreciated. Have we moved too far from the idea of the academic as expert and the student as novice?

### Note-taking

Our final foundational skill that has been the casualty of the innovative turn is note-taking. This is strange on two fronts. First, note-taking is a skill in and of itself that was long deemed necessary to teach (see Robin et al. 1977). Second, note-taking is an important active, even if not socially interactive, skill.

This skill has undoubtedly also fallen prey to technological developments and the replacement of pen and pencil with keyboard. It may, therefore, be doubly necessary to teach to a generation of students who have spent a good deal of their education to date free from a pen and paper. (Are we alone in sitting through dissertation supervision meetings where you insist a student writes something down and they have to borrow pen and paper to do so?) This has been further exacerbated by university efforts geared towards increasing student satisfaction (lecture capture and slides), which directly work against the pedagogical imperative of nudging students towards taking meaningful notes. In this section, therefore, we identify note-taking as a multi-faceted skill—when properly conceptualised.

Minute-taking is a commonly required skill in many workplaces, such that students taking electronic notes might be considered to be acquiring a skill of use in their future employment. But this is to misunderstand minute-taking. Minutes usually reflect the main points and spirit of a meeting, rather than recording what was said verbatim. In other words, they require thought, they are the distillation of the discussion and depend on discernment, the ability to categorise and prioritise. So, is the ability to type quickly sufficient? Where do we talk to students about the need to move beyond the bare recording of what was said through their electronic note-taking, to the subsequent act of distillation that the production of minutes entails?

There is another, vital, point to consider as well. Students who take notes by hand acquire a better *conceptual* understanding of a lecture than those who capture more *detail* electronically (Mueller and Oppenheimer 2014: 1159). Leaving aside employability, in any university, knowledge of facts is important. If lectures are at least partly about a transmission of facts, electronic note-taking is helpful. However, in order to excel at university, students need to acquire a good *conceptual understanding* of their subject. Mueller and Oppenheimer emphasise that students who take notes electronically rather than by hand are “hurting learning” (2014: 1166). How to address this? There is no easy method through which to bridge the divide between knowledge acquisition and conceptual understanding; both outcomes *can* be achieved *if* students revisit their typed notes, expand on them, restructure them and supplement them through reference to relevant literature and evidence.

But how can we ensure that students undertake this exercise? It is not enough to *tell* students what is better for them (Mueller and Oppenheimer 2014: 1166). Remedies are simple and multiple. To give an active demonstration, early in their degree programme, a course could require students to take notes on pen and paper and then compare perceptions of long-term recall and deep understanding in comparison to other modules where they typed notes. Assessment patterns could be adjusted to incentivise the practice of written note-taking, whereby students submit notes on lectures in classes that demonstrate both knowledge (excellent summation of lecture material) and understanding (prioritisation and analysis of information and contextualisation). In experiencing this for themselves and getting inspired by how their peers develop these skills, students are given the tools to reflect on ways of learning.

Crucially also, agency is reclaimed by both academics and students. Students are given experiential insights into pedagogy as a whole. Academics who institute such methods are now centred as experts not only in teaching but also in learning—something which students, drilled in the narratives of the “student experience” too often seem to forget.

## Concluding remarks: the need to bring back agency

We have not overlooked our own agency as academic instructors in ignoring the importance of the foundational skills of reading, listening and note-taking while promoting active learning pedagogy. We also had been somewhat seduced by the superficially portrayed employability agenda as well as the professionalisation demand from university bureaucracies. We were encouraged to focus on presenting instead of writing/reading/note-taking skills; to satisfy students by providing, effectively, our notes instead of making time to show them how to develop their own. Emphasis was on the perfect presentation, but no space was given to experience and to reflect on the need for failure or detours in academic thinking and researching. We have outlined, above, some of the structural drivers of our attachment to innovative teaching methods, but it would be disingenuous to pretend that those structural pressures were not applied to an already open door.

**Fig. 3:**
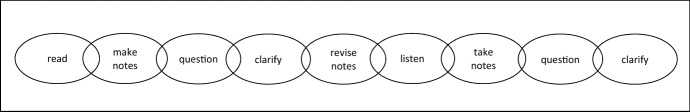
Marrying skills

Our early readings of the literature on active learning saw us thinking in terms of binaries: that we need to move *from* the traditional and passive *to* the innovative and active. A crude visualisation of the classroom serves as an illustration. In traditional teaching, the lecturer stands at the front of the classroom, the students in rows facing them. In flipped classrooms, the space is literally reconceived; thus, students are at the front of the class, the lecturer to the side or the back; in other forms of active learning, the students sit at the centre of the room, the lecturer alongside them. Thus, the students take ownership of the classroom, the lecturer relegated to the role of facilitator. Today, we would both argue there is a clear place in which the expert stands *before* the students some, but certainly not all, of the time. Thus, we are calling for a marrying of skills, as represented in Fig. 3, on the basis that active learning cannot take place in the absence of reading, listening and note-taking, all of which were more obviously accounted for in traditional learning.

Our experience in more innovative spaces was that those foundational skills were not only wrongly taken for granted but that their absence was exacerbated by many of the tools employed in the name of “teaching professionalisation” or “student satisfaction”. Carpenter and Witherby (2020), examining students’ “*illusions of learning*”, show convincingly that there is “an important disconnect between students’ impressions of effective teaching and the actual evidence of it. Students routinely associate ‘effective’ teaching with experiences that feel easy, smooth, fluent, or enjoyable”. But should learning be these things (all the time)? Who decides? And on what grounds?

Although we started thinking and writing about this prior to the pandemic, it is worth reflecting on how what we say here is impacted by the dramatically increased use of online teaching, where academics were forced to reflect on pedagogy in a way they never had to before (see the Political Studies Association’s excellent webinar series^5^). The question of academic skills, employability and active learning remained pertinent in online teaching. While the consensus seems to hold that moving traditional learning formats online alone is not sufficient, the understandable fire-fighting nature of the pandemic-necessitated changes meant there has so far been limited engagement with how academic and professional skills could be (better) integrated and consciously developed in online learning environments. A good deal of the discussions arising from the forced adoption of online teaching has been focussed on how to incorporate active learning into this space (see Political Studies Association’s webinars^5^ and also Mihai 2021). But online, as well as offline, there has been little (if any) discussion of how we encourage and ensure reading and note-taking are performed. There has been more about listening, as a product of understanding attention spans are more limited online than off—an understanding which we hope will be turned into reflection on what we might have missed when thinking about the offline environment too. As many universities look to continuing to deliver classes online throughout 2021, while others toy with the idea of instituting more blended learning in the future, our concern remains the same.

Interestingly, despite the varying institutional and national contexts in which we have taught, in our conversations over the past decade, we have been struck by the commonalities in our experiences; and, more recently, by how we have come to see the virtues of structure. However, our reflections have led us to conclude that academics must do more to ensure that the function of educational bureaucracy is to guide, not dictate; to steer, not direct; and that the voice of the academic is not lost. Bureaucracy is the structure within which academics teach and students learn but it must be shaped in such a way as to afford sufficient agency both to academics and students and play a supportive rather than restraining role. Those in the Social Sciences who are under pressure to deliver on employability must explain the negative consequences of a universalising structure on our disciplines and on our students—and give evidence on how we already do what we should and do it well. Equally, we must consider what can be improved. In organising our classrooms, we need to reconsider the place and value of the lecture, think about how to ensure foundational skills are developed *with* the student and make the arguments for teaching in the manner we think best suited for the purposes *we* define. In short, we need to reclaim the academy.

## Footnotes

1 We are grateful to one of our reviewers for this point.

2 See American Democracy Program: https://www.aascu.org/programs/ADP/.

3 See Council of Europe: https://www.coe.int/en/web/higher-education-and-research/publications.

4 While strictly speaking there is a difference between note-making (what we hear) and note-taking (what we read) (Kirton and McMillan 2007), we use note-taking as the overall term.

5 Political Studies Association Teaching and Learning Webinar Series **‘**Teaching Politics and IR Online: Key Issues, Skills and Pedagogy’. Available at https://www.psa.ac.uk/resources/flipping-covid-19-classroom, accessed 01 August 2021.
